# The polyamidoamine-mediated inhibition of bcl-2 by small hairpin RNA to induce apoptosis in human lens epithelial cells

**Published:** 2012-01-12

**Authors:** Xin-Hua Wu, Yi Lu, Yan-Wen Fang, Yong-Xiang Jiang

**Affiliations:** Department of Ophthalmology, Eye and ENT Hospital, Fudan University, Shanghai, China

## Abstract

**Purpose:**

To investigate whether apoptosis of human lens epithelial cells (HLECs) can be induced with the polyamidoamine (PAMAM)**-**mediated inhibition of *bcl-2* (b-cell lymphoma 2) by small hairpin RNA (shRNA).

**Methods:**

HLECs (SRA01/04) were transfected with the fifth generation of PAMAM (PAMAM G5) by *bcl-2* shRNA. At 24, 48, and 72 h after transfection, the transfection rate was measured by flow cytometry. The transfection rates mediated by PAMAM and liposome were compared. The *bcl-2* mRNA level was detected by real-time PCR. Whole cell protein was extracted and the bcl-2 protein level was detected by western blotting. The percentage of HLECs undergoing apoptosis was measured by Annexin V-FITC/PI staining. The nuclear morphology of HLECs was observed by staining with Hoechst 33258. The expression of cytochrome c and the activity of cleaved caspase-3 were analyzed by western blotting.

**Results:**

At 24, 48, and 72 h after transfection, the rate of transfection of *bcl-2* shRNA mediated by PAMAM was higher than in the liposome-mediated group (p<0.05). The mRNA and protein levels of *bcl-2* were greatly downregulated. The percentage of HLECs undergoing apoptosis was greatly improved. Hoechst staining showed that *bcl-2* shRNA transfected cells had a lower growth status with nuclear fragmentation. The expression of cytochrome c and the activity of cleaved caspase-3 was greatly improved (p<0.05).

**Conclusions:**

PAMAM-mediated *bcl-2* shRNA can downregulate the expression of *bcl-2* and induce the apoptosis of HLECs by engaging the mitochondrial pathway, including catalytic activation of the caspases.

## Introduction

Posterior capsule opacification (PCO) is the most common complication of cataract surgery causing visual impairment. The main cause of PCO is the proliferation, migration, and metaplasia of remnant lens epithelial cells (LECs) after cataract surgery [1-3]. Approaches for the prevention of PCO, such as the improvement of surgical techniques and intraocular lens (IOL) design and the use of antiproliferative drugs, have greatly decreased the PCO rate. However, the occurrence is still significant, at about 8%–34.3% in adults and nearly 100% in children [4-8]. Therefore, the development of an alternative therapy for preventing PCO is of critical importance. Gene therapy provides a novel strategy for the treatment of PCO; however, the present methods have drawbacks in terms of efficiency and safety [9-11].

RNA interference (RNAi) has been one of the most exciting discoveries of the past few years. Since its discovery, RNAi has shown great potential as a gene therapy [12,13]. In our previous study, we successfully constructed two pairs of *bcl-2* (b-cell lymphoma 2) small hairpin RNAs (shRNAs) and confirmed that both of them, transfected by liposome, could inhibit the expression of *bcl-2* in human LECs (HLECs) [14]. However, the low transfection efficiency of liposome has limited its application.

Polyamidoamine (PAMAM) dendrimers have received a great deal of attention as a new class of gene carriers over the past few years [15,16]. PAMAM is a nonvirus, nanometer-sized carrier with the advantages of high transfection efficiency and long-term stable expression without immunogenicity or genotoxicity. In the present study, we used the fifth generation of PAMAM (PAMAM G5) to transfect LECs and compare its efficiency with liposome, the common nonviral vector of gene therapy. Furthermore, we investigated the expression of cytochrome c and cleaved caspase-3 to determine whether the apoptosis was induced through the mitochondrial pathway [17,18].

## Methods

### Small hairpin RNA preparation and plasmid construction

Two pairs of shRNA were designed according to the *bcl-2* sequence in GenBank (NM_000633), mainly following Tuschl’s rules [19]. The sequence was transcribed with the DNA polymerase III U6 promoter in plasmid pGCsi purchased from GeneChem Co. Ltd. (Shanghai, China). The plasmid pGCsi carries the *GFP* (green fluorescent protein) gene. In our previous study, we confirmed that both of them could successfully inhibit the expression of *bcl-2* in HLECs [14]. In the present study, we use one of these shRNAs, called *bcl-2* shRNA. Its forward sequence is: 5′-GAT CCC AAG TGA AGT CAA CAT GCC TGC TTC AAG AGA GCA GGC ATG TTG ACT TCA CTT TTT TT-3′.

### Cell culture and transfection with the small hairpin RNA expression vector

SRA01/04 cells (a HLEC line) were cultured in DMEM medium supplemented with 10% fetal bovine serum, 2.05 mmol/l L-glutamine, 100 U/ml of penicillin and 100 μg/ml streptomycin at 37 °C, and 5% CO_2_. For transfection, the cells were seeded in six-well plates at 1×10^6^ cells/well and allowed to grow overnight to 90%–95% confluency. In the PAMAM group, they were transfected with the mixture of 2 μg plasmid DNA and 12 μg PAMAM G5 (Sigma-Aldrich, St. Louis, MO) in 2 ml serum-free medium. In the liposome group, they were transfected with a mixture of 2 μg plasmid DNA and 4 μl Lipofectamine^TM^ 2000 (Invitrogen, Carlsbad, CA) in 2 ml serum-free medium. At 6 h after transfection, the medium was replaced by normal medium containing 10% fetal bovine serum and antibiotics up to 48 h after transfection. In addition to the medium control, cells were transfected with negative control shRNA.

### Detection of transfection efficiency

At 24, 48, and 72 h after transfection, the samples were digested and centrifuged at 20,000× g for 10 min. After removing the supernatant, 200 μl of buffer was added and analyzed by flow cytometry. The difference in transfection efficiency between the PAMAM group and the liposome group at different times was compared. Transfected cells are green under a fluorescent microscope.

### Detection of *Bcl-2* mRNA expression by real-time PCR

Total RNA from HLECs was prepared using Trizol® Reagent (Invitrogen, Carlsbad, CA). cDNA synthesis was conducted according to the RNA PCR kit protocol (Takara, Dalian, China). The *GAPDH* (glyceraldehyde 3-phosphate dehydrogenase) gene was used as a normalizing control. The designed paired primers were as follows: *bcl-2*, 5′-CAT CTC TGT TTT CTG GCC ACA-3′ (forward), 5′-ATC CTC CTC AGA AAC AGC AGC-3′ (reverse); *GAPDH*, 5′-ACC ACA GTC CAT GCC ATC AC-3′ (forward), 5′-TCC ACC ACC CTG TTG CTG TA-3′ (reverse). The PCR reaction was performed in a volume of 20 µl using SYBR green mix (Takara, Dalian, China) on an MXP3000 instrument (Stratagene Laboratories, La Jolla, CA). PCR results were analyzed using Opticon Monitor Analysis 2.0 software (Bio-Rad Laboratories, Inc., Hercules, CA). Each sample was tested at least three times.

### Detection of Bcl-2 protein expression by western blot

Cells were lysed in a buffer containing 50 mmol/l Tris-HCl, pH 8.0, 150 mmol/l NaCl, 100 μg/ml phenylmethylsulfonyl fluoride, and 1% TritonX-100. Total protein in cell lysates was measured with the Bio-Rad colorimetric kit. Here, 50 μg protein lysate was separated by 12% SDS–PAGE and transferred onto polyvinylidene fluoride. After blocking in a 10% nonfat dry milk solution in washing buffer containing 10 mmol/l Tris (pH 7.6), 150 mmol/l NaCl, and 0.05% Tween-20, membranes were incubated for 1 h at room temperature with mouse monoclonal IgG1 of anti-bcl-2 (sc-7382; Santa Cruz Biotechnology, Santa Cruz, CA). Then they were washed four times and further incubated for 1 h with goat antimouse horseradish peroxidase–coupled secondary antibodies (Zhongshan Golden Bridge Biotechnology, Beijing, China) at room temperature. Signals were detected with the ECL kit (Amersham Pharmacia Biotech UK Ltd., Little Chalfont, UK). β-Actin staining served as the internal standard for all membranes.

### Apoptosis assay

Apoptotic cells were measured with an Annexin V/FITC kit (Bipec Biopharma, Cambridge, MA) according to the manufacturer's instructions and analyzed by flow cytometry at 48 h after transfection. Experiments were conducted in triplicate. Furthermore, morphological alteration of cell apoptosis in SRA01/04 was observed under a confocal microscope using Hoechst 33258 (Sigma-Aldrich) staining approach. The expression of cytochrome c and the activity of cleaved caspase-3 were investigated by western blot as mentioned above. Anti-cytochrome c (sc-13561; Santa Cruz Biotechnology, Santa Cruz, CA) and anti-cleaved caspase-3 (sc-22171; Santa Cruz Biotechnology) were used in western blot.

### Statistical analysis

All experiments were performed in triplicate. The one-way ANOVA was used to determine the statistical significance of the data obtained. The software used was SPSS 11.0 (SPSS for Windows, version 11.0, SPSS Inc., Chicago, IL). A value of p<0.05 was taken to represent a statistically significant difference between group means.

## Results

### Transfection efficiency

**Figure 1:**
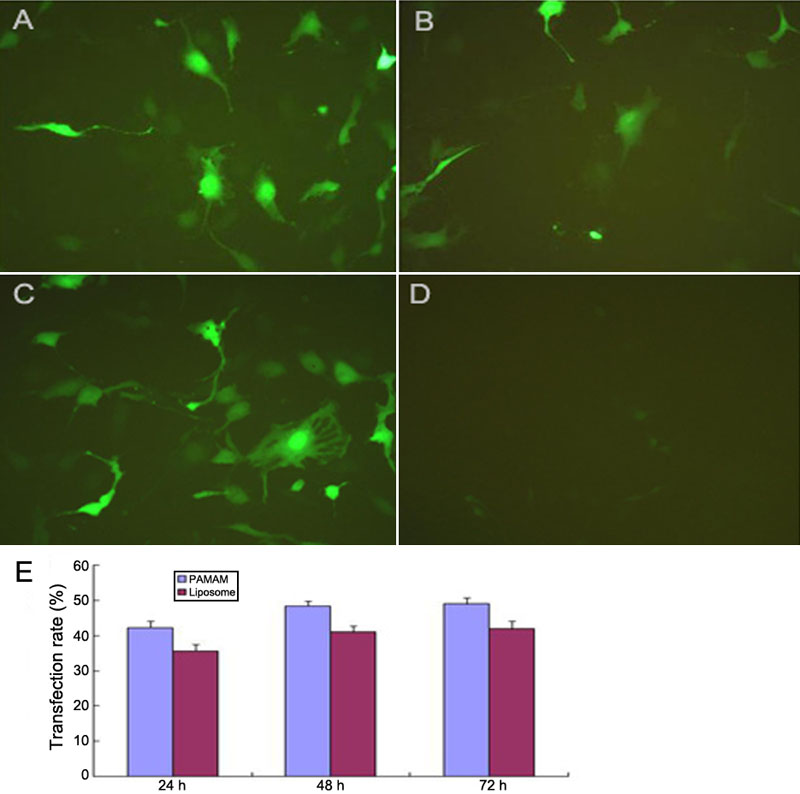
Human lens epithelial cells were transfected successfully with polyamidoamine(PAMAM) and liposome. At 48 h after transfection, green (transfected) cells could be seen in the PAMAM group (**A**), liposome group (**B**) and negative control group (**C**) under fluorescent microscope (10×20), while there was almost no green cell in the empty control group (**D**).The transfection rate of the PAMAM group and liposome group at 24, 48, and 72 h after transfection were compared (**E**).

The transfection rates of the PAMAM group and liposome group at 24, 48, and 72 h after transfection were 42.3±2.0%, 47.7±1.8%, 48.5±1.5%, and 35.1±1.8%, 41.1±1.7%, 42.0±2.3%, respectively. The difference was significant (p<0.05). We also observed the cells with a fluorescent microscope and found many green cells, which showed that they had been transfected, in the PAMAM group, liposome group, and negative control shRNA group; there were almost no green cells in the empty control group (Figure 1).

### Detection of *bcl-2* mRNA expression by real-time PCR

**Figure 2:**
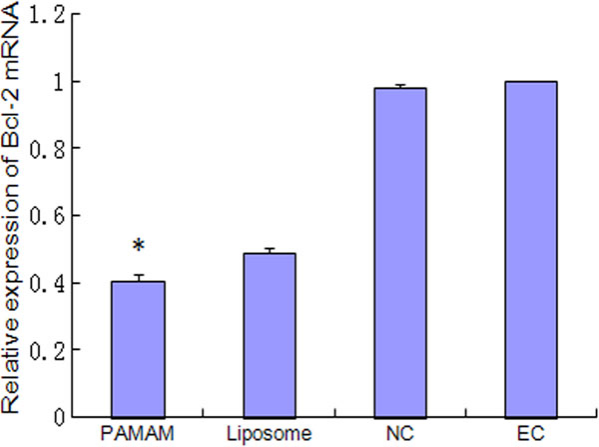
*Bcl-2* mRNA expression is suppressed by *Bcl-2* shRNA. At 48 h after transfection, the relative expression of *Bcl-2* mRNA was measured using real-time PCR. The *Bcl-2* mRNA expression of the PAMAM group and liposome group were greatly reduced compared with the negative control shRNA group (NC group) and the empty control group (EC group).* p<0.05 is significantly different from the liposome group.

Real-time PCR showed that the *bcl-2* mRNA expression of the PAMAM group and liposome group were greatly reduced compared with the negative control shRNA group and the empty control group. The expression in the PAMAM group was significantly lower than that in the liposome group (p<0.05; Figure 2).

### Detection of bcl-2 protein expression by western blot

**Figure 3:**
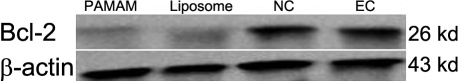
Bcl-2 protein expression is suppressed by *Bcl-2* shRNA. At 48 h after transfection, the Bcl-2 protein expression of the PAMAM group and liposome group were greatly reduced compared with the negative control shRNA group (NC group) and the empty control group (EC group).

Western blot showed that the bcl-2 protein expression of the PAMAM group and liposome group were greatly reduced compared with the negative control shRNA group (NC group) and the empty control group (EC group; Figure 3).

### Apoptosis assay

**Figure 4:**
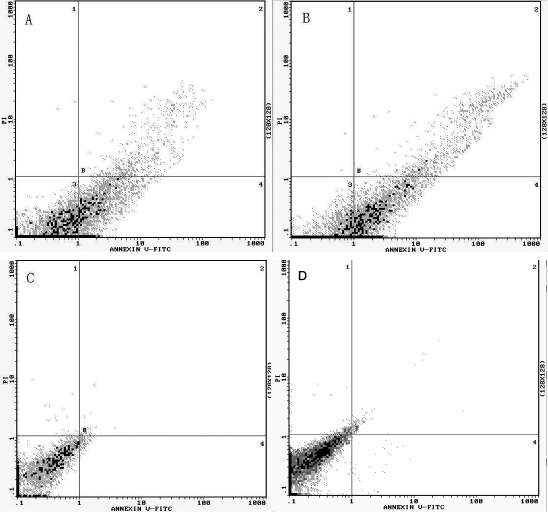
*Bcl-2* shRNA induced HLEC apoptosis. Apoptosis was measured by annexin V-FITC/PI staining and analyzed with flow cytometry. Horizontal and vertical axes represent labeling with annexin V-FITC and PI, respectively. The lower left indicates live cells; the lower right shows early apoptotic cells. The upper left shows necrotic cells, while the upper right demonstrates late apoptotic cells. The FACS plots shown were a representative of three independent experiments. The percentage of HLECs undergoing apoptosis was greatly increased in the PAMAM group (**A**) and liposome group (**B**), compared with the negative control group (**C**) and the empty control group (**D**).

**Figure 5:**
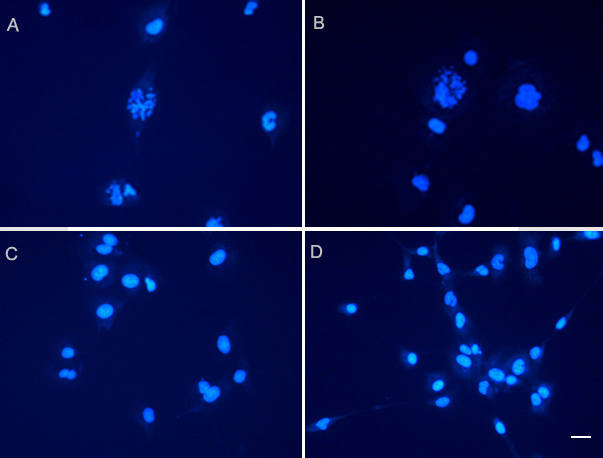
*Bcl-2* shRNA induced HLECs apoptosis. Apoptosis was observed by Hoechst 33258 staining (original magnification 40×). After human lens epithelial cells (HLECs) were transfected for 48 h, marked morphological changes were clearly seen on Hoechst 33258 staining in the PAMAM group (**A**) and the liposome group (**B**), compared with the negative control group (**C**) and the empty control group (**D**). The changes were suggestive of apoptosis, including condensation of chromatin, nuclear fragmentations, and apoptotic bodies. Scale bar, 20 µm.

**Figure 6:**
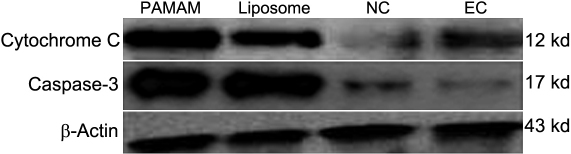
Western blot analysis of cytochrome c and cleaved caspase-3 protein. At 48 h after transfection, the cells were harvested and processed for western blotting. β-Actin was used as the internal control. The immunoblot was a representative of three independent experiments. The expression of cytochrome c and the activity of cleaved caspase-3 were greatly improved (p<0.05) in the PAMAM group and liposome group, compared with the negative control group and the empty control group.

The percentage of HLECs undergoing apoptosis, measured by annexin V-FITC/PI staining, was greatly improved in the PAMAM group and liposome group at about 45.7±1.2% and 40.9±0.8%, respectively (Figure 4). Hoechst staining showed that *bcl-2* shRNA transfected cells had a lower growth status, with nuclear fragmentation and condensation (Figure 5). The expression of cytochrome c and the activity of cleaved caspase-3 were greatly increased (p<0.05) in these two groups (Figure 6).

## Discussion

The key to preventing PCO is to eradicate the residual lens cells following surgery. In the past few years, a new strategy was proposed to remove the residual lens cells by inducing apoptosis. Previous studies have shown that Bax (bcl-2 associated X protein) or procaspase-3 overexpression is capable of inducing therapeutic programmed cell death in vitro and in vivo in residual lens cells and preventing PCO in a rabbit model [20].

Our strategy was to remove the residua lens cells by inducing apoptosis with RNAi. In our study, we approved that knockdown of *bcl-2* can induce the apoptosis of HLECs. As we know, bcl-2 is a prominent member of the bcl-2 family of proteins, which regulate the induction of apoptotic cell death by a wide variety of stimuli. Knockdown of *bcl-2* by shRNA was able to induce apoptosis in many tumor tissues [21,22]. The present investigation showed that it could also induce apoptosis in HLECs. The percentage of HLECs undergoing apoptosis, measured by annexin V-FITC/PI staining, was greatly improved in the PAMAM group and liposome group. The morphological alteration of cell apoptosis could also be observed under a confocal microscope using Hoechst 33258 staining approach. These data demonstrated that apoptosis of HLECs was induced. The results were similar to overexpress Bax or procaspase-3 using adenovirus-mediated gene transfer [20].

In our study, the expression of cytochrome c and the activity of cleaved caspase-3 were greatly increased. As we know, apoptosis is induced via two main routes involving either the mitochondria or the activation of death receptors. The mitochondrial pathway is activated by a wide range of signals and involves the release of proteins (including cytochrome c) from the mitochondrial membrane space. Cytochrome c leads to the activation of caspase-9, which then triggers a cascade of caspase activation (caspase-3, caspase-6, caspase-7), resulting in the morphological and biochemical changes associated with apoptosis [17,18]. The present investigation suggest that the apoptosis is induced by engaging the mitochondrial pathway, including catalytic activation of the caspases [20].

The difficulty of gene therapy is how to transfer the gene into the target tissue. Viral or nonviral vectors have their own usage in different conditions [23-28]. PCO is not life threatening; therefore, the use of potentially harmful viral vectors is questionable. Nonviral vectors have potential advantages. Most of them are safe and nontoxic to ocular tissues. The major drawback of nonviral vectors is their low transfection rate and relatively short life. Over the past few years, PAMAM has emerged as a new class of nanoscopic, spherical polymers that have captured the interest of researchers in various scientific disciplines [29-31]. Here, in a comparison between PAMAM and liposome, we found that PAMAM had higher transfection efficiency than liposome. Considering its safety and multifunctionality, it appears to have bright prospects in gene therapy.

In summary, we have shown that *bcl-2* shRNA can inhibit the expression of *bcl-2* in HLECs and induce cell apoptosis by engaging the mitochondrial pathway, including catalytic activation of the caspases. Further work is needed to restrict RNAi to the remaining HLECs and avoid harming neighboring normal tissues such as the cornea and iris before this approach can be used for the treatment of human disease.
